# Imaging in-vivo tau pathology in Alzheimer’s disease with THK5317 PET in a multimodal paradigm

**DOI:** 10.1007/s00259-016-3363-z

**Published:** 2016-03-21

**Authors:** Konstantinos Chiotis, Laure Saint-Aubert, Irina Savitcheva, Vesna Jelic, Pia Andersen, My Jonasson, Jonas Eriksson, Mark Lubberink, Ove Almkvist, Anders Wall, Gunnar Antoni, Agneta Nordberg

**Affiliations:** Department NVS, Center for Alzheimer Research, Division of Translational Alzheimer Neurobiology, Karolinska Institutet, Novum 5th floor, 141 57 Huddinge, Sweden; Department of Radiology, Karolinska University Hospital Huddinge, Stockholm, Sweden; Department of Geriatric Medicine, Karolinska University Hospital Huddinge, Stockholm, Sweden; Radiology, Department of Surgical Sciences, Uppsala University, Uppsala, Sweden; Medical Physics, Uppsala University Hospital, Uppsala, Sweden; PET Centre, Uppsala University Hospital, Uppsala, Sweden; Pre-clinical PET Platform, Uppsala University, Uppsala, Sweden; Department of Psychology, Stockholm University, Stockholm, Sweden

**Keywords:** Positron emission tomography, Alzheimer’s disease, Tau, Neurofibrillary tangles, THK5317, Other dementia, Non-AD, Amyloid PET, FDG, PIB

## Abstract

**Purpose:**

The aim of this study was to explore the cerebral distribution of the tau-specific PET tracer [^18^F]THK5317 (also known as *(S)*-[^18^F]THK5117) retention in different stages of Alzheimer’s disease; and study any associations with markers of hypometabolism and amyloid-beta deposition.

**Methods:**

Thirty-three individuals were enrolled, including nine patients with Alzheimer’s disease dementia, thirteen with mild cognitive impairment (MCI), two with non-Alzheimer’s disease dementia, and nine healthy controls (five young and four elderly). In a multi-tracer PET design [^18^F]THK5317, [^11^C] Pittsburgh compound B ([^11^C]PIB), and [^18^F]FDG were used to assess tau pathology, amyloid-beta deposition and cerebral glucose metabolism, respectively. The MCI patients were further divided into MCI [^11^C]PIB-positive (*n* = 11) and MCI [^11^C]PIB-negative (*n* = 2) groups.

**Results:**

Test-retest variability for [^18^F]THK5317-PET was very low (1.17–3.81 %), as shown by retesting five patients. The patients with prodromal (MCI [^11^C]PIB-positive) and dementia-stage Alzheimer’s disease had significantly higher [^18^F]THK5317 retention than healthy controls (*p* = 0.002 and *p* = 0.001, respectively) in areas exceeding limbic regions, and their discrimination from this control group (using the area under the curve) was >98 %. Focal negative correlations between [^18^F]THK5317 retention and [^18^F]FDG uptake were observed mainly in the frontal cortex, and focal positive correlations were found between [^18^F]THK5317 and [^11^C]PIB retentions isocortically. One patient with corticobasal degeneration syndrome and one with progressive supranuclear palsy showed no [^11^C]PIB but high [^18^F]THK5317 retentions with a different regional distribution from that in Alzheimer’s disease patients.

**Conclusions:**

The tau-specific PET tracer [^18^F]THK5317 images in vivo the expected regional distribution of tau pathology. This distribution contrasts with the different patterns of hypometabolism and amyloid-beta deposition.

**Electronic supplementary material:**

The online version of this article (doi:10.1007/s00259-016-3363-z) contains supplementary material, which is available to authorized users.

## Introduction

Alzheimer’s disease (AD) in vivo diagnosis has been reconceptualised since the introduction of amyloid-beta-specific PET tracers. Amyloid-beta accumulation becomes apparent early in the asymptomatic stage of the disease [1], however, it reaches a plateau when the first symptoms occur [2], preventing further monitoring of the disease progression. Abnormal tau hyperphosphorylation and deposition also start early [3] but are known to better relate to disease severity and cognitive decline [4], and represent, thus, an attractive marker for assessing the temporal evolution of the disease.

The wide spectrum of degenerative tauopathies includes AD, corticobasal degeneration, progressive supranuclear palsy etc. Those diseases are associated with different isoforms of tau protein as well as different regional distribution of the underlying pathology [5]. Therefore, PET imaging of the load and regional distribution of tau pathology in vivo constitutes a great challenge for the research on neurodegenerative diseases.

Several families of promising tau-specific tracers have been developed during the last years, with few in-vivo reports published [6–8]. Among these tracers, THK5117 has shown high affinity for and selective binding to tau pathology [9, 10], and its *S*-form enantiomer (also known as [^18^F]THK5317) showed favourable pharmacokinetics [11]. However, there remains a paucity of evidence regarding imaging tau pathology in different stages of AD as well as other tauopathies.

Until recently, the study of the regional distribution of tau pathology in relation to amyloid-beta deposition and neurodegeneration was only possible *post-mortem*. However, *post-mortem* studies are limited to a single time point, most often at the end stage of the disease, and provide only a weak link between the individual’s *ante-mortem* clinical profile and the underlying pathology. The combination of imaging markers for amyloid-beta and cerebral metabolism with the newly developed tau tracers is opening exciting new opportunities for the in-vivo research of AD and other tauopathies.

The aims of this study were two-fold: 1) using [^18^F]THK5317-PET, to evaluate the presence and regional distribution of tau pathology in vivo in a cohort of AD patients; and 2) to examine the regional distribution of tau pathology in relation to hypometabolism and amyloid-beta deposition in vivo.

## Methods

### Study population

The 33 enrolled individuals included nine patients diagnosed with AD (57–75 years old), 13 with mild cognitive impairment (MCI) (59–79 years old), four age-matched elderly healthy controls (eHC; 58–71 years old) and five young healthy controls (yHC; 20–30 years old). Two patients with non-AD dementia were also recruited, in an exploratory manner, including one with a clinical diagnosis of corticobasal degeneration and one with progressive supranuclear palsy. All patients were referred to the Memory Clinic at the Department of Geriatric Medicine, Karolinska University Hospital, Stockholm, Sweden, and underwent thorough clinical investigation including medical history, physical examination, laboratory blood tests, Apolipoprotein E (ApoE) genotyping, neuropsychological assessment, cerebrospinal fluid sampling and MRI scans. The diagnosis was based on the consensus of a committee, which included geriatricians, neurologists, clinical neuropsychologists, and specialist nurses.

All AD patients fulfilled the National Institute of Neurological and Communication Disorders, Alzheimer’s Disease and Related Disorders Association criteria [12] and the DSM-IV criteria for Dementia of the Alzheimer’s type [13], while all MCI patients met the Petersen criteria [14]. Eleven MCI patients were classified as amnestic single-domain, and two as amnestic multi-domain [15]. All amnestic single-domain MCI patients were classified as MCI PIB-positive (*n* = 11) based on their amyloid status, as determined by their [^11^C]Pittsburgh compound B (PIB) PET scan (see the “[^11^C]PIB- and [^18^F]FDG-PET image pre-processing” section below), while the amnestic multi-domain MCI patients were classified as MCI PIB-negative (*n* = 2). For the purposes of this project, all MCI PIB-positive patients were reclassified into a prodromal AD group in accordance with the new research diagnostic criteria [16]. Similarly, all PIB-positive patients with clinically diagnosed AD were reclassified into the AD dementia group. This resulted in eleven patients with prodromal AD, nine with AD dementia and two with MCI PIB-negative.

The two patients with non-AD dementia were clinically diagnosed as follows: one patient with predominantly left-sided signs of atypical parkinsonism, progressive apraxia and executive deficits fulfilled the criteria for possible corticobasal degeneration syndrome [17], and one patient with progressive postural instability and falls, vertical supranuclear opthalmoparesis, dysdiadochokinesia and executive deficits fulfilled the criteria for probable progressive supranuclear palsy [18].

Nine individuals (five yHC and four eHC) were recruited as healthy controls either via Clinical Trial Consultants AB (Uppsala University Hospital, Uppsala, Sweden) or from patients’ familial circles, after extensive clinical evaluation. They were included in the absence of cognitive complaint, prior head injury, or known neurologic/psychiatric disorder. They were all non-smokers and free from medication.

### Neuropsychological assessment

All individuals completed a large battery of neuropsychological tests. Individual performances (except mini mental state examination (MMSE) performance) are expressed as z-scores, from comparison with a reference group of healthy controls [19].

Global cognition was assessed as the average performance in nine tests, including five subtests from the Weschler Adult Intelligence Scale (i.e. Similarities, Information, Block Design, Digit Span, and Digit Symbol), the Corsi test, the Trail-Making Tests A and B, and the Rey-Osterrieth complex figure copy test. Episodic memory was assessed as the average performance in the following three tests: the Rey-Auditory Verbal Learning learning and 30 min delayed-retention tests, and the Rey-Osterrieth complex figure retention subtest.

### Image acquisition

A PET scan using [^18^F]THK5317 and a 3D-T1-weighted MRI sequence were acquired for all participants. Additionally, all participants except the yHC group underwent a [^11^C]PIB-PET scan, and all patients (i.e. those with prodromal AD, AD dementia, MCI PIB-negative, and non-AD dementia) underwent an [^18^F]FDG-PET scan.

The [^18^F]THK5317- and [^11^C]PIB-PET scans were acquired on an ECAT EXACT HR+ scanner (Siemens/CTI) or a Discovery ST PET/CT scanner (GE) at the Uppsala PET centre, Uppsala, Sweden. Both tracers were synthesised according to standard good manufacturing processes as previously described [11, 20]. For [^18^F]THK5317-PET, 22 frames were acquired over 60 min (6 × 10 s, 3 × 20 s, 2 × 30 s, 2 × 60 s, 2 × 150 s, 4x300 s, and 3x600 s frames) after intravenous injection of 212 ± 42 MBq. For [^11^C]PIB-PET, 24 frames were acquired over 60 min (4 × 30s, 9 × 60 s, 3 × 180 s and 8 × 300 s) after intravenous injection of 253 ± 69 MBq. The [^18^F]FDG-PET scans were acquired on a Biograph mCT PET/CT scanner (Siemens) at the Department of Nuclear Medicine, Karolinska University Hospital Huddinge, Stockholm, Sweden, with a 15 min static run, 30 min after injection (30–45 min) of 3 MBq/kg. All acquisitions were reconstructed using ordered subset expectation maximisation.

### [^18^F]THK5317-PET image pre-processing

All T1-weighted MRI images were divided into grey and white matter (GM and WM, respectively) tissue classes using SPM8 software’s unified segmentation. The inverse non-linear transformation from this segmentation step was used to wrap a probabilistic atlas [21] from MNI space into each individual’s native T1 space. The resulting individual atlases were subsequently multiplied using the corresponding binarised probabilistic GM mask, to obtain individual GM atlases. In addition to 30 bilateral regions of interest (ROI) in the probabilistic atlas, two composite bilateral GM ROIs were created, according to the classical neuropathological staging of tau pathology [22]: a limbic ROI (Braak stages III–IV) comprising the following regions: hippocampi, amygdalas, and parahippocampal, fusiform, middle inferior temporal, orbital and straight frontal gyri, as well as temporal poles and parietal-temporal-occipital junctions; and an isocortical ROI (Braak stages V–VI), comprising all isocortical regions except the precentral and postcentral gyri.

Individual dynamic [^18^F]THK5317-PET images were co-registered onto the individual T1-weighted image with PMOD v.3.5 software (PMOD Technologies Ltd., Adliswil, Switzerland). In order to create distribution volume ratio (DVR) images, the reference Logan graphical method was applied to the [^18^F]THK5317 images over the 30–60 min scan interval, with cerebellar GM—extracted from the T1 segmentation—as a reference, as previously described [11].

### Additional [^18^F]THK5317 preprocessing methods

Standard uptake value (SUV) images (40–60 min) were also created for [^18^F]THK5317. The SUV was defined as the radioactivity concentration (MBq/mL) divided by [injected dose (MBq)/ patient’s weight (kg)]. Standard uptake value ratio (SUVR) images, using cerebellar GM—extracted from the T1 segmentation—as a reference, were generated for all individuals. Additional SUV images were used to assess the patient with progressive supranuclear palsy, since the latter disease is characterised by the presence of tau pathology in the cerebellum [23].

The [^18^F]THK5317 dynamic images were also analysed, using the Muller-Gartner partial volume correction method as implemented in PMOD v.3.5 software, based on the individual T1-weighted images.

### [^11^C]PIB- and [^18^F]FDG-PET image pre-processing

[^11^C]PIB-PET (40–60 min) and [^18^F]FDG-PET (30–45 min) images were created and co-registered onto the individual T1-weighted images using SPM8. SUVR images were created using the cerebellar GM as reference for [^11^C]PIB and the pons for [^18^F]FDG scans. An SUVR threshold of 1.41 for amyloid positivity was applied to [^11^C]PIB-PET data [24]. Additional DVR images for [^11^C]PIB retention were generated using the reference Logan graphical method over the 30–60-min scan interval, with cerebellar GM as a reference, as previously described for [^18^F]THK5317 DVR.

### Test-retest

Five patients (four with prodromal AD and one with corticobasal degeneration) underwent a second PET scan with [^18^F]THK5317 on a separate day, not more than 37 days from the first. DVR images were created as previously described.

### Statistical analysis — ROI-based comparisons

Differences between all healthy controls (i.e. yHC and eHC), prodromal, and dementia-stage AD for continuous variables were assessed with the non-parametric Kruskal–Wallis one-way analysis of variance (ANOVA), with post-hoc Mann–Whitney Bonferroni-corrected pairwise comparisons; medians and interquartile ranges (IQR) are reported. In detail, to assess comparisons across the three diagnostic groups in the two composite ROIs (limbic and isocortical), Bonferroni-corrected alpha levels of 0.008 were applied (0.05/6 [3 groups × 2 ROIs]). Fisher’s exact tests were used to assess differences between groups for discrete variables.

Within-individual test-retest variability was assessed using the relative difference [(Retest-Test)/Test] in retention between scans in ROIs from the probabilistic atlas. The intraclass correlation coefficient, a measure of reproducibility was applied.

In order to determine which ROIs from the probabilistic atlas were best for discriminating between AD patients (i.e. patients with prodromal and AD dementia) and all healthy controls (i.e. yHC and eHC), receiver operating characteristic (ROC) analysis was performed and the area under the curve (AUC) was determined for each ROI.

All the above-mentioned statistical comparisons were carried out using SPSS v.22.0 software (Armonk, NY: IBM Corp) for Mac OS X. Graphical representations were carried out with the ggplot2 package v.1.0.1 as implemented in R v.3.1.3 software.

Correlation matrices—incorporating the Pearson coefficient—were created to evaluate, two by two, the cortical ROI-based relationships between [^18^F]THK5317 DVR retention and [^18^F]FDG SUVR uptake, using the Hmisc v.3.14 and corrplot v.0.73 packages as implemented in R v.3.1.3 software.

### Statistical analysis — Voxel-based comparisons

Comparisons of [^18^F]THK5317 DVR retention among all healthy controls (i.e. yHC and eHC), prodromal AD, and AD dementia patients at the voxel-level were assessed, two by two, using a non-parametric alternative to two-sample T-tests. Due to the limited sample sizes of each diagnostic group significant differences were assessed with a standard non-parametric procedure based on permutation testing, as implemented in the statistical non-parametric mapping toolbox (SnPM13) [25]. [^18^F]THK5317 DVR images were registered into the MNI space using the corresponding transformation matrix obtained at the T1 segmentation step, and were smoothed (FWHM = 8 mm in all directions) using SPM8. Statistical significance was assessed with permutation tests with 10,000 permutations corrected for multiple comparisons using the false discovery rate test (*p* < 0.05).

Individual retention in patients was also compared with the mean retention in the healthy controls with the creation of individual z-score maps. For [^18^F]THK5317, mean and standard deviation images were created from the DVR images from the five yHCs. A z-score threshold of 1.96 (95 % confidence interval) was used to determine areas of abnormally high [^18^F]THK5317 retention (z-scores > 1.96). [^18^F]THK5317 DVR images from each patient were binarised according to the threshold, and were consequently summed in order to illustrate the areas of abnormally high retention in each diagnostic group (Online Resource 1).

Relationships between tracers with respect to retention at the voxel-level were assessed, two by two, with the BPM (Biological Parametric Mapping) 3.1 toolbox [26], using the individual PET images registered into the MNI space and smoothed, as discussed above. A cluster threshold of 20 voxels was applied, with no correction for multiple comparisons (*p* < 0.001). Two more lenient thresholds (*p* < 0.01 and *p* < 0.05) were also applied to acknowledge the limited sample size.

The results of the voxel-based comparisons were projected onto either the individual or the group average cortical surfaces using Freesurfer 5.3 (http://surfer.nmr.mgh.harvard.edu/).

## Results

### Study population

The characteristics of the study population are summarised in Table 1. There were no statistically significant differences between prodromal AD and AD dementia groups with regard to age or gender. Patients with prodromal AD and AD dementia only differed significantly with respect to their neuropsychological assessment; patients with dementia scored lower on the MMSE test and the composite measure of episodic memory than the prodromal patients.

**Table 1:** Demographic and clinical characteristics of the study population

	yHC	eHC	MCI PIB-positive (Prodromal AD)	AD dementia	PSP	CBD	MCI PIB-negative
*n*	5	4	11	9	1	1	2
Age, years	22 [22:28]	62 [60.3:65]	73 [65:74]	67 [60:74]	66	69	64 ; 79
Gender (male/female)	0/5	3/1	5/6	2/7	1/0	1/0	1/1
ApoE ε4 frequency	n/a	n/a	6 (60 %)^†^	7 (78 %)	0 (0 %)	n/a	0 (0 %)^†^
Education, years	15 [14.3:17]	14 [12.5:15.5]	12 [10:14]	14 [12:15]	9	15	8 ; 18
MMSE	n/a	n/a	29 [27.5:29.5] ^a^	24 [23:25]	24	23	23 ; 27
Global cognition, z-scores	0.9 [0.9:1.2]	−0.6 [−1.1:0.0]	−0.5 [−0.8:0.2]	−2.5 [−6.3:0.3] ^b^	−3.3	−3.3	−1.0 ; −1.5
Episodic memory, z-scores	1.0 [0.7:1.2]	−0.7 [−1.2:0.7]	−1.5 [−1.9:0.1] ^a^	−2.6 [−2.8:-2.0] ^b^	n/a	−2.0	−1.0 ; −0.9
Time PIB to THK5317, days	n/a	0 [0–2]	0 [0–4]	0 [0–7]	0	9	0 ; 0
Time FDG to THK5317, days	n/a	n/a	56 [38:84]	60 [41:64]	106	92	101 ; 620
Time CSF to THK5317, days	n/a	n/a	745 [492:993]	857 [391:1888]	103	694	558 ; 636
CSF Aβ_1–42_, _pg/mL_	n/a	n/a	547 [506.3:851]^§^	484.5 [449.8:526.5]^†^	693	1700	607 ; 792
CSF t-tau, _pg/mL_	n/a	n/a	465 [301:492]^‡^	546 [368:675.8]^†^	239	644	210 ; 933
CSF p-tau_181p_, _pg/mL_	n/a	n/a	62.5 [49.8:86.5]^§^	82 [57:104.3]^†^	36	98	36 ; 120

Significant differences with a Bonferroni-corrected threshold of *p* < 0.016 for pair-wise comparisons ^a^relative to patients with AD dementia; ^b^relative to all HC (i.e. yHC and eHC)

Data are presented as medians [Interquartile range], or as n(%). *Aβ* amyloid-beta, *AD* Alzheimer’s disease, *ApoE* apolipoprotein E, *CBD* corticobasal degeneration, *eHC* elderly healthy controls, *FDG* [^18^F]FDG, *MMSE* mini mental state examination, *n*/*a* results not available, *PIB* [^11^C]Pittsburgh compound B, *MCI PIB*-*positive*/*negative* mild cognitive impairment with [^11^C]PIB uptake above/below the normal range (threshold standard uptake value ratio of 1.41), *PSP* progressive supranuclear palsy, *THK5317* [^18^F]THK5317, *yHC* young healthy controls

^†^data missing for one individual

^‡^data missing for three individuals

^§^data missing for four individuals

### Test-retest

There was low within-individual variability in the [^18^F]THK5317 DVR values (Table 2). The mean absolute relative difference ranged from 1.17 to 3.29 % for the isocortical ROIs and from 1.51 to 3.81 % for the subcortical ROIs. The intraclass correlation coefficient ranged from 0.86 to 0.98 for the isocortical ROIs and from 0.94 to 0.98 for the subcortical ROIs, with only the posterior cingulate cortex resulting in a coefficient of 0.52.

**Table 2:** Test (T)-retest (R) comparison for [^18^F]THK5317 DVR regional values in five patients

Individual	Region of Interest										
	Frontal cx	Temporal cx	Parietal cx	Occipital cx	Insular cx	Anterior cingulate gyrus	Posterior cingulate gyrus	Hippocampus	Putamen	Caudate nucleus	Thalamus
Prodromal AD n^o^ 1 – T	1.20	1.16	1.06	1.08	1.29	1.24	1.25	1.32	1.67	1.08	1.34
Prodromal AD n^o^ 1 – R	1.20	1.14	1.05	1.08	1.26	1.22	1.25	1.30	1.66	1.08	1.35
% Difference	−0.47	−1.81	−0.93	0.18	−2.03	−1.16	0.31	−1.26	−0.26	0.41	1.04
Prodromal AD n^o^ 2 – T	1.13	1.11	1.10	1.20	1.22	1.23	1.21	1.30	1.46	0.89	1.27
Prodromal AD n^o^ 2 – R	1.15	1.13	1.09	1.16	1.20	1.26	1.26	1.37	1.51	1.05	1.34
% Difference	2.29	1.74	−0.39	−2.91	−2.30	2.79	4.21	5.36	2.93	18.92	5.38
Prodromal AD n^o^ 3 – T	1.24	1.14	1.06	1.17	1.31	1.23	1.23	1.44	1.63	1.31	1.42
Prodromal AD n^o^ 3 – R	1.24	1.15	1.05	1.18	1.32	1.28	1.29	1.48	1.62	1.39	1.48
% Difference	−0.20	0.71	−0.59	0.81	1.17	3.68	4.80	3.02	−0.70	5.72	4.43
Prodromal AD n^o^ 4 – T	1.17	1.08	1.11	1.10	1.26	1.25	1.28	1.33	1.59	1.00	1.38
Prodromal AD n^o^ 4 – R	1.19	1.10	1.11	1.11	1.29	1.25	1.25	1.30	1.63	0.99	1.38
% Difference	1.98	1.42	0.05	0.27	2.86	0.52	−1.84	−2.65	2.07	−0.58	−0.04
CBD – T	1.09	1.10	1.05	1.08	1.23	1.14	1.21	1.19	1.79	0.74	1.20
CBD – R	1.08	1.06	1.01	1.03	1.24	1.11	1.15	1.11	1.76	0.74	1.18
% Difference	−1.07	−3.45	−3.90	−3.98	0.36	−2.29	−5.28	−6.75	−1.58	−0.70	−1.81
Mean absolute, % Difference	1.20	1.83	1.17	1.63	1.74	2.09	3.29	3.81	1.51	5.27	2.54
Standard deviation	0.91	1.00	1.56	1.71	0.98	1.27	2.12	2.21	1.07	7.96	2.27
Intraclass correlation coefficient	0.98	0.86	0.90	0.95	0.90	0.93	0.52	0.94	0.98	0.97	0.95

*AD* Alzheimer’s disease, *cx* cortex, *CBD* corticobasal degeneration, *cx* cortex, *Prodromal AD* mild cognitive impairment with [^11^C]PIB uptake above the normal range (threshold standard uptake value ratio of 1.41); % Difference: [(R-T)/T]; Intraclass correlation coefficient: [(BIMSS−WIMSS)/(BIMSS + WIMSS)], where *BIMSS* = between-individuals mean sum of squares, and *WIMSS* = within-individual mean sum of squares. Frontal, temporal, parietal and occipital cortices were defined according to the Hammers’ probabilistic atlas [21]

### [^18^F]THK5317 retention and regional distribution in AD

**Fig. 1:**
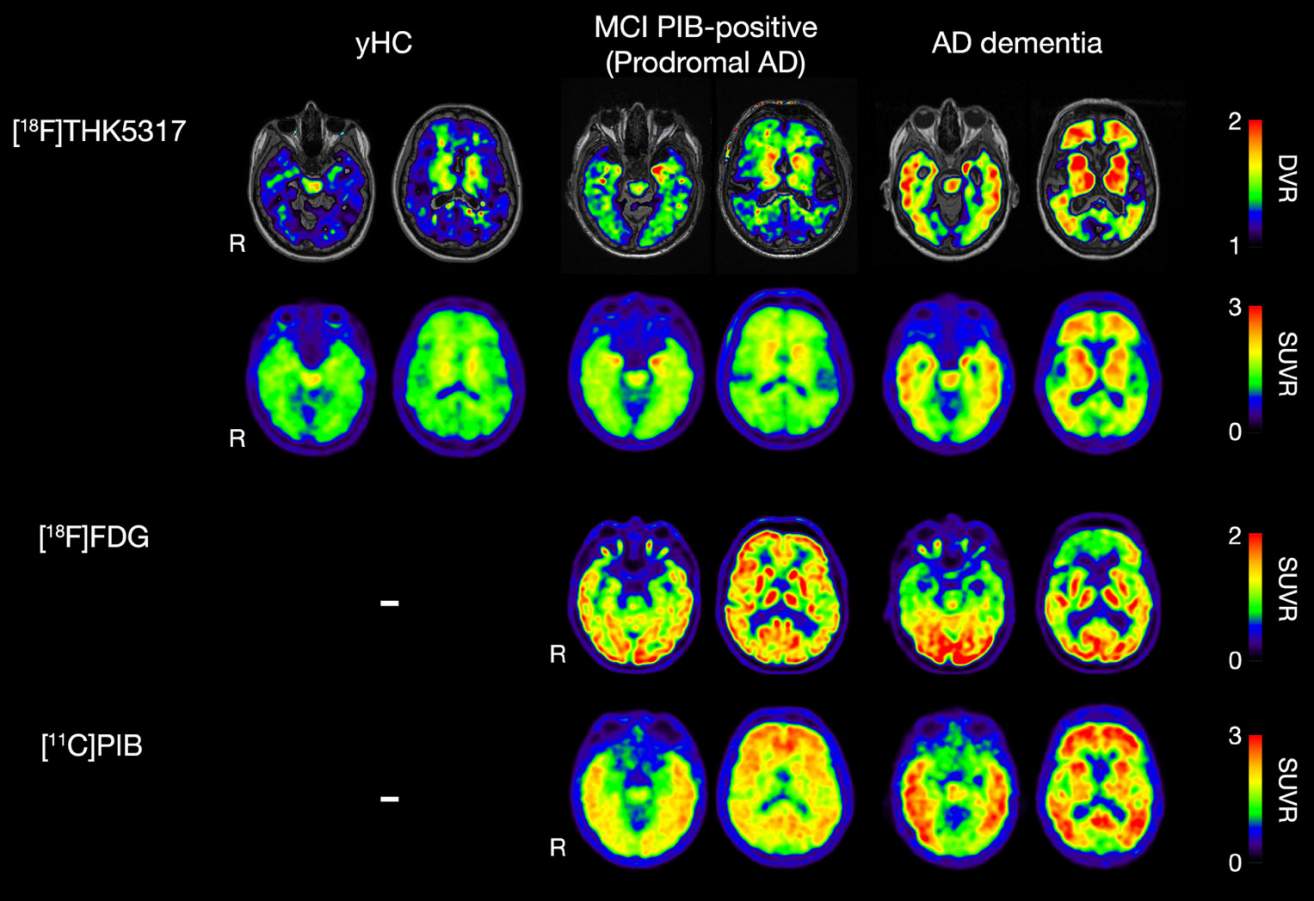
Sample [^18^F]THK5317 DVR, and [^18^F]THK5317, [^18^F]FDG and [^11^C]PIB SUVR images from a young healthy control (yHC), and patients with prodromal and dementia-stage Alzheimer’s disease (AD). DVR = distribution volume ratio; PIB = [^11^C]PIB; Prodromal AD = mild cognitive impairment (MCI) with [^11^C]PIB retention above the normal range (threshold standard uptake value ratio of 1.41); R = Right; SUVR = standard uptake value ratio

[^18^F]THK5317 retention was high in both prodromal and AD dementia patients in isocortical and subcortical regions, in direct contrast to the lack of retention in the yHC group (Fig. 1). [^18^F]FDG uptake was low in isocortical areas in AD patients, with more pronounced changes in the dementia group.

**Fig. 2:**
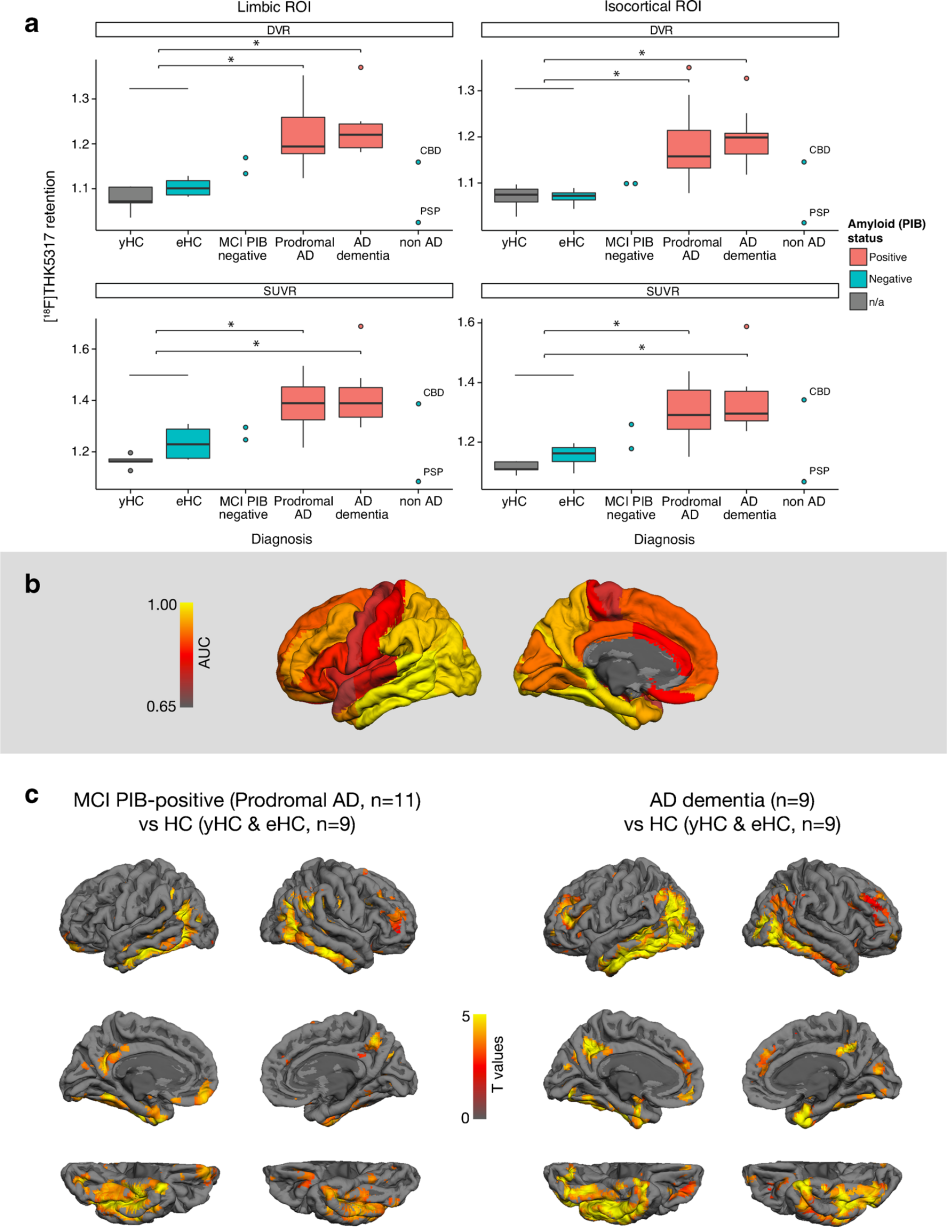
[^18^F]THK5317 retention in and discrimination ability between healthy controls and patients with Alzheimer’s disease (AD). The boxplots represent [^18^F]THK5317 DVR and SUVR retention across diagnostic groups in the composite limbic and isocortical regions of interest (ROIs) (**a**). The color-coding represents the area under the curve (AUC) of the individual cortical ROIs for discrimination between all healthy controls (i.e. yHC and eHC) and all AD patients (i.e. prodromal and dementia-stage) (**b**). Voxel-based comparisons of [^18^F]THK5317 DVR retention (SnPM) among all healthy controls (i.e. yHC and eHC), prodromal AD, and AD dementia patients after correction for multiple comparisons with the use of the false discovery rate test (*p* < 0.05) (**c**). The threshold for [^11^C]PIB positivity = 1.41. Filled dots represent outliers and individuals belonging to the MCI [^11^C]PIB-negative and non AD groups; eHC = elderly healthy controls; PIB = [^11^C]PIB; Prodromal AD = mild cognitive impairment (MCI) with [^11^C]PIB retention above the normal range (threshold standard uptake value ratio of 1.41); non AD = non-AD dementia; yHC = young healthy controls; n/a = not available; * *p* < 0.05

The composite limbic and isocortical [^18^F]THK5317 (DVR, SUVR) retention across the diagnostic groups is illustrated in Fig. 2a. There was no overlap in [^18^F]THK5317 DVR retention between AD dementia patients and all healthy controls (i.e. yHC and eHC), and almost no overlap between prodromal AD patients and healthy controls. No significant differences in [^18^F]THK5317 retention were observed between prodromal and AD dementia patients. A similar pattern of [^18^F]THK5317 retention, but with a wider range and greater overlap between diagnostic groups, was observed when using SUVR quantification (Figs. 1, and 2a).

Good discrimination between the different diagnostic groups was seen in most ROIs (Online Resource 2). Discrimination between all AD patients (i.e. patients with prodromal and AD dementia) and all healthy controls (i.e. yHC and eHC) was best in the composite limbic ROI, the isocortical ROI, and the temporal cortex, with AUC values of 0.994, 0.983, and 0.978, respectively, followed by the occipital (0.958), parietal (0.944), and frontal (0.872) cortices (Fig. 2b). In the subcortical ROIs, the AUC in the putamen reached 0.853.

Voxel-based comparisons between all healthy controls (i.e. yHC and eHC) and patients with prodromal as well as AD dementia revealed widespread neocortical areas with increased [^18^F]THK5317 retention in both patient groups (Fig. 2c), including predominantly the inferior and lateral temporal, anterior frontal, lateral occipital, and inferior parietal cortices, as well as the precuneus. No significant difference was observed in [^18^F]THK5317 retention between prodromal and AD dementia patients across the neocortex.

**Fig. 3:**
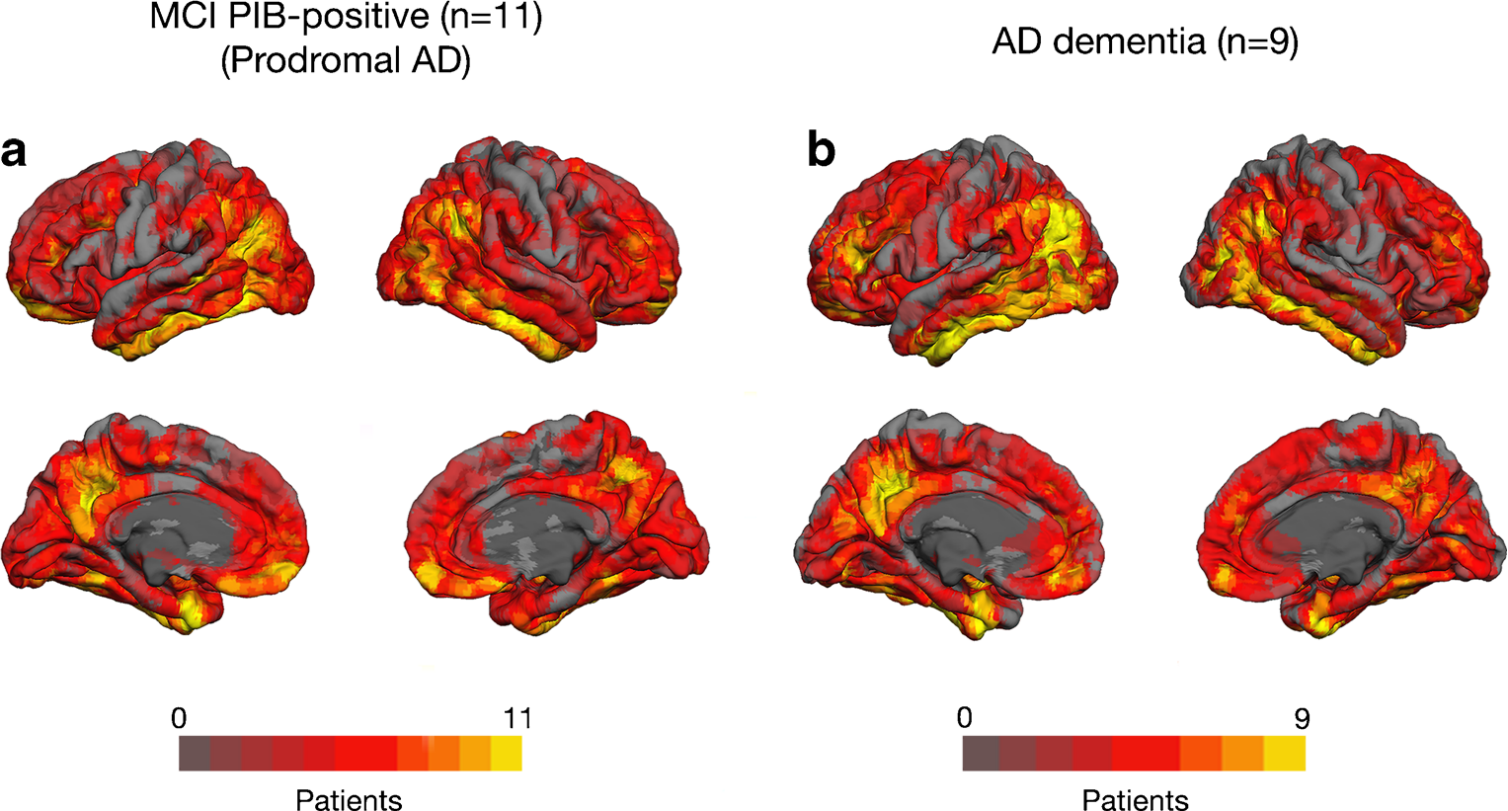
Regional patterns of abnormal [^18^F]THK5317 retention across all patients with prodromal Alzheimer’s disease (AD) (*n* = 11) and AD dementia (*n* = 9). Individual [^18^F]THK5317 DVR images were compared to those from the five young healthy controls using z-score maps. Only voxels with z-score values above 1.96 (95 % confidence interval) were considered. The resulting individual binarised images were summed to illustrate the areas of abnormal retention in the two diagnostic groups (Online Resource 1). DVR = distribution volume ratio; PIB = [^11^C]PIB; SUVR = standard uptake value ratio

In comparison to the yHC group only, [^18^F]THK5317 retention was higher in the AD patients in areas similar to the ones mentioned above (Fig. 3a–b). The primary motor, somatosensory, and superior temporal cortices appeared to be spared from high [^18^F]THK5317 retention. Although a similar pattern of [^18^F]THK5317 retention was observed in the patients with prodromal and AD dementia, abnormally high [^18^F]THK5317 retention in the lateral temporal, lateral occipital, and parietal regions was observed in greater proportion in AD dementia patients. Of note, similar findings were found to a large extent when using the eHC group as a reference (Online Resource 3).

### Correlations between the regional distributions of the different tracers

**Fig. 4:**
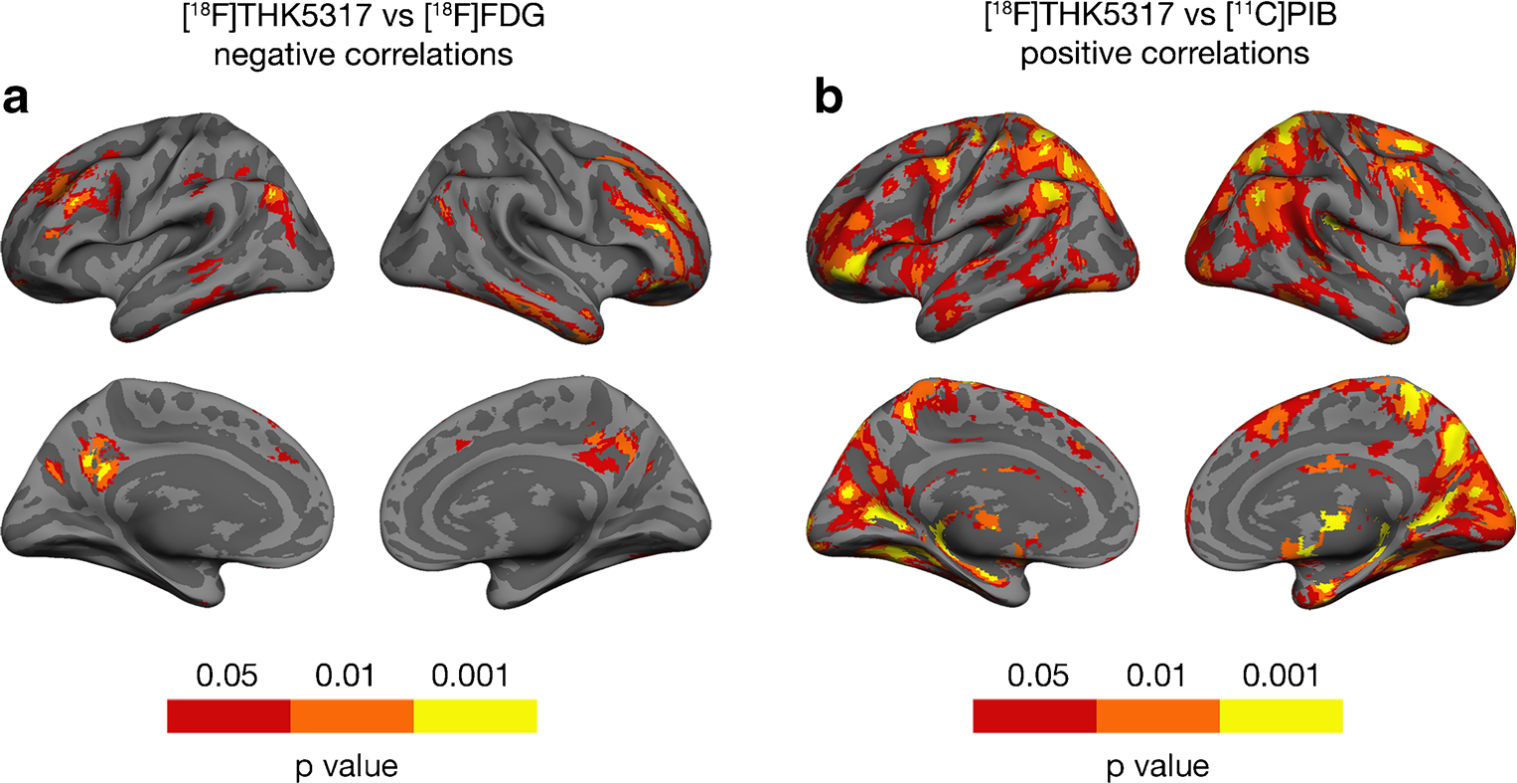
Correlations between tracers, two by two, across all Alzheimer’s disease patients (*n* = 20). Voxel-based negative correlations between [^18^F]THK5317 DVR retention and [^18^F]FDG SUVR uptake (**a**). Voxel-based positive correlations between [^18^F]THK5317 DVR and [^11^C]PIB SUVR retention (**b**). Three thresholds for statistical significance were applied (*p* < 0.001, *p* < 0.01, and *p* < 0.05) as indicated. The peak voxels of clusters meeting the *p* < 0.001 threshold are detailed in Online Resource 4 and 6. DVR = distribution volume ratio; SUVR = standard uptake value ratio

Across all AD patients (*n* = 20), at a voxel level, significant negative correlations between [^18^F]THK5317 DVR retention and [^18^F]FDG SUVR uptake were observed in focal areas of the prefrontal cortex and the lateral and mesial parietal cortices unilaterally (*p* < 0.001) (Fig. 4a, Online Resource 4). More liberal thresholds for significance (*p* < 0.01 and *p* < 0.05) revealed more extensive bilateral areas of the same regions as well as significant correlations in the lateral temporal cortex. Positive correlations (*p* < 0.001) between the two tracers were observed bilaterally in the superior temporal (max *T* = 9.9), occipital (max *T* = 7.7), and primary motor (max *T* = 8.9) cortices. In the ROI-based comparisons only negative correlations were observed between the regional retention of the two tracers; predominantly in the frontal cortex (Online Resource 5).

[^18^F]THK5317 DVR retention positively correlated with [^11^C]PIB SUVR retention (*p* < 0.001) bilaterally, in the mesial and inferior temporal, anterior and superior frontal, lateral and mesial parietal, and occipital cortices (Fig. 4b, Online Resource 6). More liberal thresholds for significance (*p* < 0.01 and *p* < 0.05) detected more extensive areas of the same regions. A similar pattern of positive correlations were observed between [^18^F]THK5317 DVR with [^11^C]PIB DVR retention (Online Resource 7). No significant negative correlations (*p* < 0.001) were observed between the two tracers.

### Elderly healthy controls

All the eHCs were found to be amyloid negative, according to their [^11^C]PIB-PET scans. Incidental MRI findings included one eHC with mesial temporal atrophy (score 1–2 out of 4) (Online Resource 8c) and one eHC with parietal atrophy (score 1–2 out of 3) (Online Resource 8d). Overall, the eHCs exhibited low [^18^F]THK5317 retention although, at an individual level, focal areas of high retention were observed in mesial areas of the frontal, temporal, and parietal cortices, in comparison to yHCs (Online Resource 8).

### Regional distribution of tau pathology in other tauopathies

The two patients with non-AD dementia were found to be amyloid-negative based on their isocortical [^11^C]PIB retention.

**Fig. 5:**
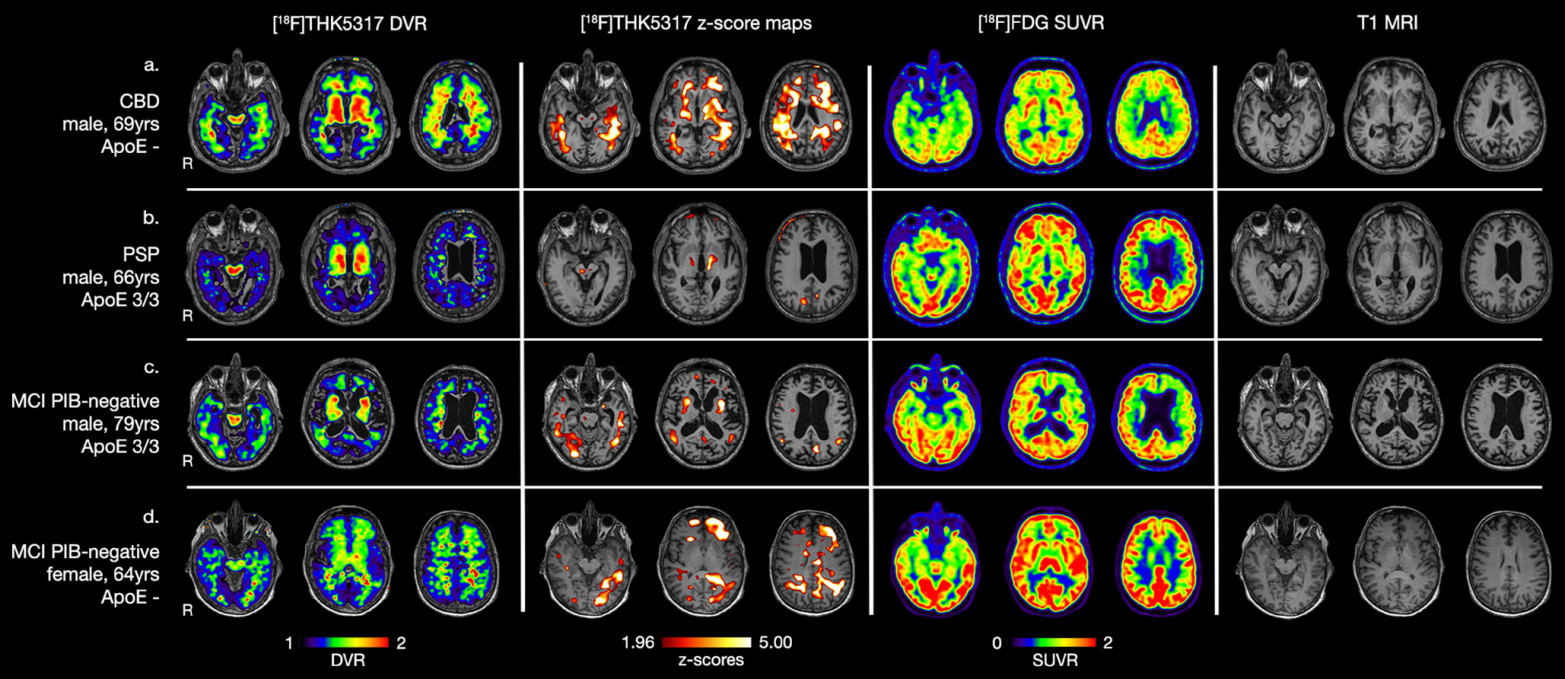
[^18^F]THK5317 DVR retention, [^18^F]THK5317 DVR z-score maps in comparison to young healthy controls (left two columns), [^18^F]FDG SUVR uptake (third column) and T1 MRI (fourth column) in the patients with non-Alzheimer’s disease dementia (first two rows) and the MCI PIB-negative patients (last two rows). A z-score threshold of 1.96 (95 % confidence interval) was applied. CBD = corticobasal degeneration; DVR = distribution volume ratio; MCI PIB-negative = mild cognitive impairment (MCI) with [^11^C]PIB uptake below the normal range (threshold standard uptake value ratio of 1.41); PIB = [^11^C]PIB; PSP = progressive supranuclear palsy; R = Right; SUVR = standard uptake value ratio

The patient with the clinical diagnosis of possible corticobasal degeneration syndrome showed widespread high [^18^F]THK5317 retention predominantly in WM areas, the basal ganglia and the WM of the cerebellum (Fig. 5a). Low [^18^F]FDG uptake, predominantly in the right hemisphere, was reported from clinical visual assessment in the parietal and prefrontal cortices, with asymmetrical uptake in the thalami. Parietal atrophy (score 1 out of 3) was observed bilaterally in the T1 MRI of the patient with a light rightward asymmetry.

**Fig. 6:**
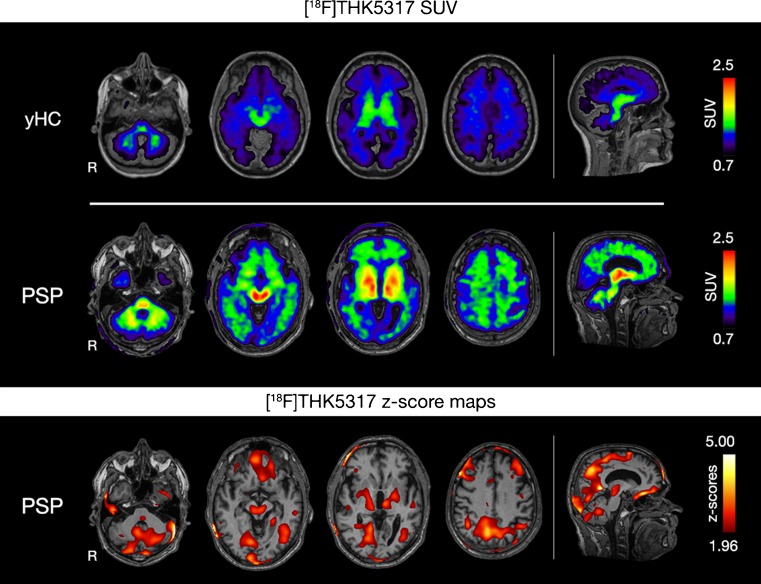
[^18^F]THK5317 SUV retention in a representative young healthy control (yHC) and a patient with progressive supranuclear palsy (PSP) (upper two rows), [^18^F]THK5317 SUV z-score map in comparison to yHCs for the PSP patient (last row). A z-score threshold of 1.96 (95 % confidence interval) was applied. R = Right; SUV = standard uptake value as the radioactivity concentration (MBq/mL) divided by [injected dose (MBq)/ patient’s weight (kg)].

The patient with the clinical diagnosis of probable progressive supranuclear palsy showed high [^18^F]THK5317 DVR retention only in the basal ganglia and the midbrain bilaterally (Fig. 5b). SUV images were created to control for possible cerebellar [^18^F]THK5317 retention. Areas of high [^18^F]THK5317 SUV retention were detected in widespread isocortical areas, as well as the basal ganglia, the brainstem and the cerebellum (Fig. 6). Areas of low [^18^F]FDG uptake in the insula, anterior cingulate cortex as well as the basal ganglia and thalami were clinically reported. Global cortical atrophy (score 1–2 out of 3) was observed on the T1 MRI of the patient.

The two MCI PIB-negative patients exhibited different patterns of high [^18^F]THK5317 retention. The first patient, a male with signs of atypical parkinsonism, progressive executive and memory deficits, showed high [^18^F]THK5317 retention predominantly in the mesial parietal cortex, the basal ganglia, and focal changes in the WM of the cerebellum, in comparison to yHC (Fig. 5c). The same patient showed a strongly leftward asymmetrical cortical pattern of low [^18^F]FDG uptake with accompanying low uptake in the basal ganglia and thalami. Global cortical atrophy (score 2 out of 3) and mesial temporal atrophy (score 3 [right] and 4 [left] out of 4) were observed on the T1 MRI of the patient. In the other MCI PIB-negative patient, a female with progressive executive deficits including word-finding difficulties, although average isocortical bilateral [^11^C]PIB retention was below the 1.41 SUVR threshold, areas of abnormally high retention were observed isocortically. Strongly asymmetrical high [^18^F]THK5317 retention was observed in the left fronto-parieto-temporal hemisphere of this patient in comparison to yHC. No areas of low [^18^F]FDG uptake or atrophy were clinically reported in this patient (Fig. 5d).

### Partial volume correction

Partial volume correction was used to account for the WM retention of [^18^F]THK5317, and the resulting spill-over from WM to GM. This resulted in a wider range of DVR values in all ROIs; e.g. in the isocortical ROI, DVR retention ranged from 1.12 to 1.71 after correction, while it ranged from 1.03 to 1.37 without correction. Most comparisons were not affected by the correction. However, the differences between all healthy controls and AD patients were greater after correction of the [^18^F]THK5317 data than before correction in ROIs prone to WM spill-over. These regions included the hippocampus, anterior cingulate, and superior temporal ROIs. The results of these comparisons are illustrated in Online Resource 9.

## Discussion

Imaging of in-vivo tau pathology will both shed new light on whether it is tau or amyloid pathologies that drive the cascade of events leading to AD, and serve as a tool for studying the progression of the disease. The key strength of this study was the use of [^18^F]THK5317, a tau-specific PET tracer with well-characterised kinetic properties [11] in a cohort of patients at different stages of AD, as well as the use of different quantification methods. The multimodal study of tau pathology, in relation to glucose metabolism ([^18^F]FDG) and amyloid-beta deposition ([^11^C]PIB), provides a suitable design for exploring the regional and temporal evolution of the underlying pathologies in AD.

### Test-retest reproducibility

The test-retest reproducibility was very high and the within-individual variability was low in the procedures using [^18^F]THK5317. The poor test-retest reproducibility in the posterior cingulate gyrus (0.52), despite the low within-individual variability (3.29 %), can be explained by the low retention variability in that ROI among the patients that underwent test-retest investigation, which negatively biased the measures of reproducibility [27].

### [^18^F]THK5317 retention in AD

The high [^18^F]THK5317 retention in the AD patients and the low retention in the healthy controls resulted in excellent discrimination between the groups in regions associated with the accumulation of tau pathology in AD. More specifically, the ranking of areas that discriminated best between all healthy controls and patients with AD strongly resembles the stereotypical pattern of the temporal spreading of tau pathology [22, 28].

Patients with prodromal AD or AD dementia in our study exhibited a regional distribution of abnormally high [^18^F]THK5317 retention that spread isocortically beyond the limbic areas [22], with no statistical significant difference observed in [^18^F]THK5317 retention between the two patient groups. It seems possible, therefore, that the isocortical propagation of tau pathology precedes the dementia-stage of the disease. Nevertheless, the AD dementia patients showed, in greater proportion than prodromal patients, abnormal [^18^F]THK5317 retention in areas that are expected to be affected late by tau pathology [22, 28]. The recruitment of mainly mild dementia patients in our study could preclude more extensive differences in [^18^F]THK5317 retention between prodromal and dementia patients.

Striatal [^18^F]THK5317 retention was observed in all clinical groups, including healthy controls, in accordance with post-mortem data [10]. This has also been reported with other newly developed tau tracers [29, 30] and is presently a source of discussion. However, in light of the good discrimination between controls and AD patients using [^18^F]THK5317 retention in the putamen, and despite the moderate non-specific retention in this region in the yHC group, it could conceivably be hypothesised that [^18^F]THK5317 is able to successfully detect underlying tau pathology in this region [31].

### Glucose metabolism and amyloid-beta deposition in relation to [^18^F]THK5317 retention

There was a negative regional correlation between [^18^F]FDG uptake and [^18^F]THK5317 retention in AD patients, although this was restricted to focal areas of the prefrontal cortex and the precuneus, suggesting that the first signs of hypometabolism occur when the tau pathology has already extended beyond the limbic region and has spread isocortically. Surprisingly, we also observed a few positive correlations in the voxel-wise comparisons in the isocortical areas spared from hypometabolism even at the latest stages of AD. This finding might be due to the use of different scanners for the two tracers. Indeed, ROI-based comparisons, less prone to noise in comparison to voxel-based analysis, identified only negative correlations between [^18^F]FDG uptake and [^18^F]THK5317 retention; predominantly in the frontal cortex.

Positive correlations were observed between [^18^F]THK5317 and [^11^C]PIB retention in these patients, suggesting the possible temporal proximity of the build-up of the two pathological processes (amyloid and tau). Moreover, it is possible that this association, regardless of the different regional distribution of the pathologies in AD, could partly reflect the co-localisation of the two hallmarks in neuritic plaques, namely dense amyloid plaques surrounded by dystrophic neurites often containing tau pathology [32]. Those significant findings, however, were not corrected for multiple comparisons and further studies need to be carried out in order to address our statistical power issues and validate our results. An additional uncontrolled factor is the possibility that the newly developed tau-specific PET tracers bind to amyloid deposits with lower affinity than tau fibrils since both consist of beta-sheet structures of protein aggregates.

### [^18^F]THK5317 retention in other tauopathies

The two patients with non-AD dementia and the two MCI PIB-negative patients showed regional [^18^F]THK5317 retention that significantly differed from the typical pattern observed in AD patients. The patient with clinically diagnosed corticobasal degeneration syndrome exhibited high retention in areas consistent with the reported neuropathological distribution of tau pathology in this disease [33], similarly to findings reported with a different tau-specific tracer [29]. The patient with clinically diagnosed progressive supranuclear palsy showed [^18^F]THK5317 retention in the expected isocortical and subcortical regions [23]. However, the regional retention patterns described in these two patients should be interpreted with caution given the clinical and pathological heterogeneity of these diseases, as well as the overlaps between them [34, 35].

The cases of non-AD pathology discussed here illustrate the complexities of tau imaging. Firstly, the clinical syndromes do not correlate well with the neuropathological findings, as discussed above. Secondly, the underlying pathology could negate the use of the cerebellum or the brainstem as reference regions. Finally, the involvement of different types of tau deposits and tau isoforms challenges the interpretation of the observed findings, since much uncertainty exists regarding the form of tau the newly developed tracers target, and future in-vitro work in pathologically confirmed cases will be of great importance.

The MCI PIB-negative patient with signs of atypical parkinsonism showed a retention pattern similar to that observed in the non-AD patients. The MCI PIB-negative patient with progressive executive deficits including word-finding difficulties showed a clearly asymmetrical fronto-parieto-temporal pattern of [^18^F]THK5317 retention. Taking the clinical and imaging profiles together, it could be speculated that this patient could demonstrate an early syndrome in the spectrum of primary progressive aphasias [36], although the patient did not meet the existing criteria at the time of the study [37]. Clinical follow-up of these two patients will reveal more information regarding their underlying pathology.

### Tracer characteristics

Several studies have documented that different optical isomers possess different pharmacological profiles [38] and that it might be best to use an enantiomerically pure compound as a tracer. The choice of the *S*-form enantiomer of the tracer [^18^F]THK5117 used in this study was based on preliminary findings suggesting it has faster kinetics and lower non-specific retention than the racemic mixture (Victor Villemagne, unpublished data). Indeed, improved pharmacokinetics and a faster dissociation rate from the WM have already been observed for the *S*-enantiomers of two other tracers of the same family [39, 40]. The *S*-form of the tracer THK5117 ([^18^F]THK5317) showed fast kinetics [11] and lower non-specific retention as visually observed in comparison to the previous reports on the racemic mixture [9, 39]. For instance, in our study clear discrimination between healthy controls and patients was observed in the putamen, a region prone to non-specific spill-over; these results are in contrast to those of an earlier study that used the racemic form of [^18^F]THK5117, where no difference was seen between groups [9].

[^18^F]THK5317 retention measured as DVR and SUVR discriminated well between all healthy controls and patients with AD, although the SUVR values showed greater overlap between groups. Taken together with previous findings indicating that SUVR values were not particularly accurate for measuring [^18^F]THK5317 retention [11], these results suggest that SUVR retention values should be interpreted with caution.

In order to account for the potential spill-over of [^18^F]THK5317 retention from WM to GM, and for the atrophy observed in the brains of patients with AD, additional analyses were carried out with partial volume correction. This resulted in roughly similar discrimination between the groups in the majority of regions. Nonetheless, after correction, better discrimination between groups was revealed in the hippocampus and anterior cingulate gyrus. This finding has important implications for future studies aiming to investigate [^18^F]THK5317 retention in those regions, given their significance in the deposition of tau pathology in AD.

### Limitations

The small sample size, especially of patients with pathologies other than AD, limits the generalisability of our findings. Moreover, only linear relationships between the different pathologies were assessed, although it is likely that non-linear models would depict best the association between biomarkers. Lastly, associations between the tracers’ retention were only assessed in corresponding regions, precluding findings between remote regions’ retention. These matters deserve to be addressed in future studies.

## Conclusion

This study shows that [^18^F]THK5317, a tau-specific PET tracer, can image with a high reproducibility the expected extent and regional distribution of tau pathology in a cohort of healthy controls and patients at different clinical stages of AD. These results indicate that tau pathology, hypometabolism, and amyloid-beta deposition follow different patterns of regional and temporal spread. Future studies incorporating cognitive measures and longitudinal design will provide a better understanding of the cascade of events leading to AD.

## Electronic supplementary material

Below is the link to the electronic supplementary material.

ESM 1 (DOCX 5507 kb)
