# Supplementary material for: Imaging in-vivo tau pathology in Alzheimer’s disease with THK5317 PET in a multimodal paradigm
Source: Eur J Nucl Med Mol Imaging. 2016 Mar 21;43:1686–99. doi: 10.1007/s00259-016-3363-z (PMC4932128; doi:10.1007/s00259-016-3363-z)
Supplement: Supplementary file 1 — (DOCX 5507 kb) [file 259_2016_3363_MOESM1_ESM.docx]

**Online Resources**

**Title:** Imaging *in vivo* tau pathology in Alzheimer’s disease with THK5317 PET in a multimodal paradigm.

**Authors:** Konstantinos Chiotis, MD,^1^; Laure Saint-Aubert, PhD,^1^; Irina Savitcheva, MD, PhD,^2^; Vesna Jelic, MD, PhD,^3^; Pia Andersen, MD,^3^; My Jonasson,^4,5^; Jonas Eriksson, PhD,^6,7^; Mark Lubberink, PhD,^4^; Ove Almkvist, PhD,^1,3,8^; Anders Wall, PhD,^4,6^; Gunnar Antoni, PhD,^6,7^; Agneta Nordberg, MD, PhD,^1,3.^

**Affiliations:** ^1^Dept NVS, Center for Alzheimer Research, Translational Alzheimer Neurobiology, Karolinska Institutet, Stockholm, Sweden; ^2^Department of Radiology, Karolinska University Hospital Huddinge, Stockholm, Sweden; ^3^Department of Geriatric Medicine, Karolinska University Hospital Huddinge, Stockholm, Sweden; ^4^Radiology, Department of Surgical Sciences, Uppsala University, Uppsala, Sweden; ^5^Medical Physics, Uppsala University Hospital, Uppsala, Sweden; ^6^PET Centre, Uppsala University Hospital, Uppsala, Sweden; ^7^Pre-clinical PET Platform, Uppsala University, Uppsala, Sweden; ^8^Department of Psychology, Stockholm University, Stockholm, Sweden.

**Corresponding Author:** Agneta Nordberg, MD, PhD, professor

Karolinska Institutet,

Dept. NVS, Center for Alzheimer Research,

Division of Translational Alzheimer Neurobiology,

Novum 5th floor, 141 57 Huddinge, Sweden

Mail: Agneta.K.Nordberg@ki.se

Phone: +46 8 585 854 67; Fax: +46 8 585 854 70


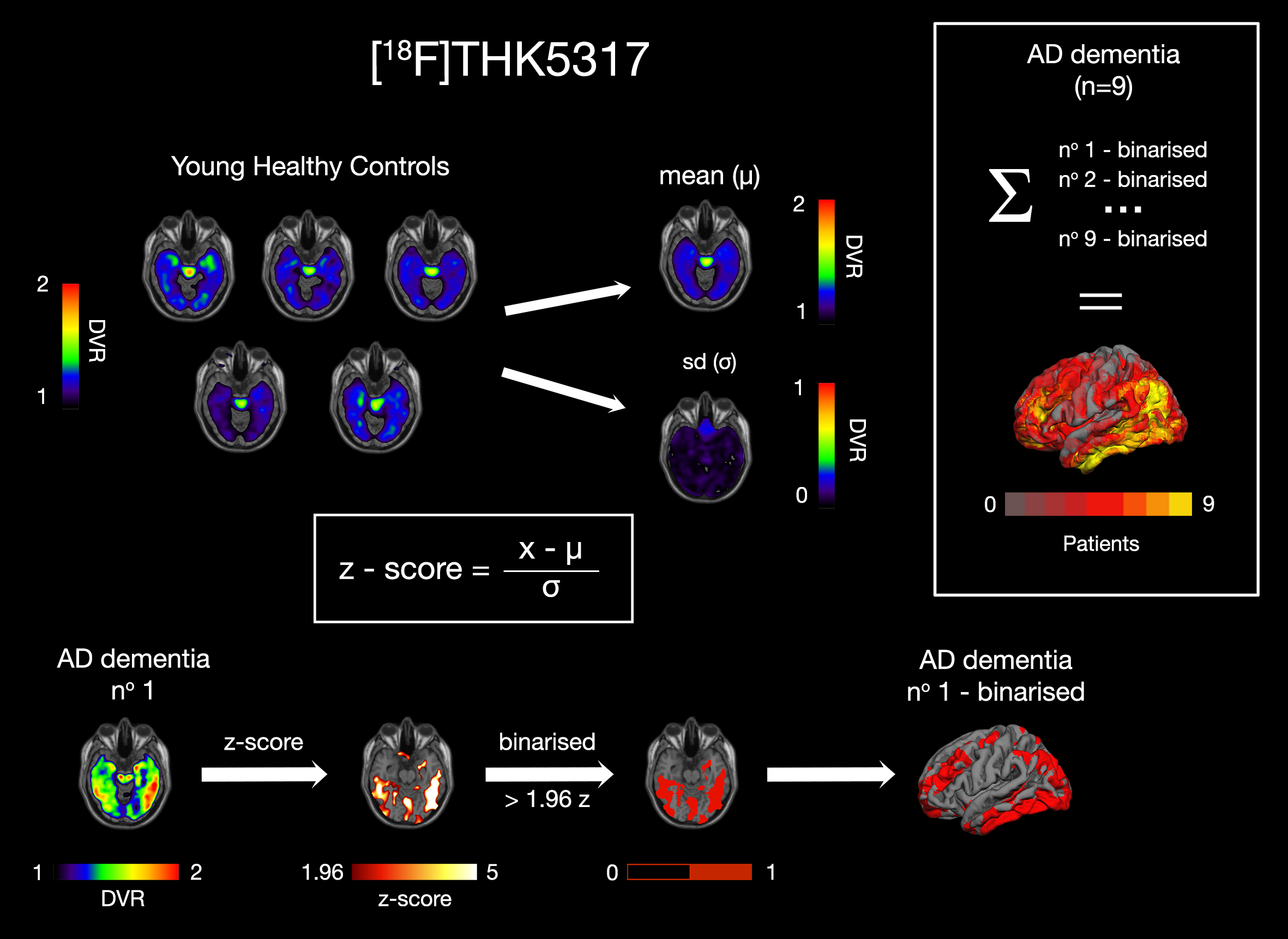


Online Resource 1. Voxel-based comparisons of [^18^F]THK5317 DVR retention in patients with Alzheimer’s disease dementia in comparison with young healthy controls using individual z-score maps. AD = Alzheimer’s disease; DVR = distribution volume ratio; sd = standard deviation.

Online Resource 2. Group differences in [^18^F]THK5317 DVR retention in the regions of interest.

| [^18^F]THK5317 DVR retention | | | |
| --- | --- | --- | --- |
| Regions of Interest | Healthy controls  (i.e. yHC and eHC)  (n=9) | MCI PIB-positive  (Prodromal AD)  (n=11) | AD dementia  (n=9) |
| Limbic | 1.09 [1.07:1.11] | 1.19 [1.16:1.26] | 1.22 [1.19:1.25] |
| Isocortical | 1.07 [1.05:1.09] | 1.16 [1.13:1.21] | 1.20 [1.16:1.23] |
| Hippocampus | 1.27 [1.25:1.36] | 1.42 [1.30:1.44] | 1.29 [1.21:1.37] |
| Amygdala | 1.28 [1.24:1.39] | 1.50 [1.40:1.62] | 1.53 [1.41:1.67] |
| Parahippocampal gyrus | 1.08 [1.04:1.10] | 1.19 [1.15:1.24] | 1.21 [1.18:1.24] |
| Fusiform gyrus | 1.08 [1.05:1.13] | 1.21 [1.18:1.29] | 1.22 [1.17:1.24] |
| Inferior temporal gyrus | 1.06 [1.05:1.09] | 1.23 [1.17:1.29] | 1.24 [1.23:1.27] |
| Anterior temporal lobe (inf) | 0.99 [0.97:1.06] | 1.11 [1.05:1.22] | 1.16 [1.12:1.18] |
| Anterior temporal lobe (med) | 1.04 [1.01:1.06] | 1.13 [1.08:1.26] | 1.16 [1.14:1.21] |
| Superior temporal gyrus (cen) | 1.10 [1.03:1.15] | 1.17 [1.12:1.23] | 1.17 [1.10:1.19] |
| Superior temporal gyrus (ant) | 0.98 [0.93:1.06] | 1.01 [1.00:1.14] | 1.03 [1.00:1.11] |
| Posterior temporal lobe | 1.09 [1.07:1.10] | 1.22 [1.16:1.30] | 1.24 [1.19:1.28] |
| Inferior frontal gyrus | 1.07 [1.04:1.09] | 1.14 [1.08:1.16] | 1.15 [1.12:1.19] |
| Anterior orbital gyrus | 1.11 [1.03:1.14] | 1.19 [1.15:1.25] | 1.23 [1.19:1.25] |
| Medial orbital gyrus | 1.09 [1.08:1.14] | 1.22 [1.17:1.25] | 1.20 [1.17:1.25] |
| Lateral orbital gyrus | 1.02 [0.97:1.05] | 1.08 [1.03:1.11] | 1.11 [1.07:1.16] |
| Posterior orbital gyrus | 1.16 [1.11:1.18] | 1.23 [1.20:1.30] | 1.26 [1.20:1.32] |
| Straight frontal gyrus | 1.17 [1.12:1.20] | 1.28 [1.25:1.31] | 1.21 [1.15:1.29] |
| Middle frontal gyrus | 1.04 [1.00:1.06] | 1.14 [1.09:1.22] | 1.18 [1.16:1.29] |
| Superior frontal gyrus | 1.03 [0.98:1.05] | 1.09 [1.04:1.15] | 1.11 [1.08:1.24] |
| Precentral gyrus | 1.02 [0.99:1.06] | 1.06 [1.04:1.12] | 1.08 [0.99:1.14] |
| Anterior cingulate | 1.22 [1.21:1.25] | 1.27 [1.23:1.31] | 1.27 [1.24:1.32] |
| Postcentral gyrus | 1.01 [0.98:1.04] | 1.05 [1.02:1.06] | 1.07 [1.01:1.11] |
| Lateral parietal lobe | 1.03 [1.02:1.07] | 1.15 [1.10:1.21] | 1.17 [1.11:1.25] |
| Superior parietal gyrus | 1.04 [1.02:1.06] | 1.16 [1.09:1.21] | 1.20 [1.10:1.24] |
| Posterior cingulate | 1.19 [1.16:1.23] | 1.28 [1.23:1.34] | 1.32 [1.25:1.40] |
| Lateral occipital lobe | 1.03 [1.02:1.06] | 1.18 [1.11:1.25] | 1.21 [1.15:1.25] |
| Lingual gyrus | 1.05 [1.04:1.09] | 1.21 [1.09:1.24] | 1.16 [1.13:1.19] |
| Cuneus | 1.05 [1.03:1.10] | 1.16 [1.10:1.21] | 1.16 [1.11:1.22] |
| Insular lobe | 1.25 [1.21:1.28] | 1.31 [1.26:1.33] | 1.28 [1.23:1.35] |
| Putamen | 1.55 [1.52:1.59] | 1.63 [1.59:1.72] | 1.70 [1.61:1.75] |
| Thalamus | 1.43 [1.34:1.50] | 1.35 [1.32:1.51] | 1.29 [1.23:1.35] |

Data are presented as medians [Interquartile range] or as p values (significance at p<0.05). AD = Alzheimer’s disease; DVR = distribution volume ratio; eHC = elderly healthy controls; MCI PIB-positive = mild cognitive impairment with [^11^C]PIB uptake above the normal range (threshold standard uptake value ratio of 1.41); ns = not significant; PIB=[^11^C]PIB; Prodromal AD = mild cognitive impairment with [^11^C]PIB uptake above the normal range (threshold standard uptake value ratio of 1.41); yHC = young healthy controls.


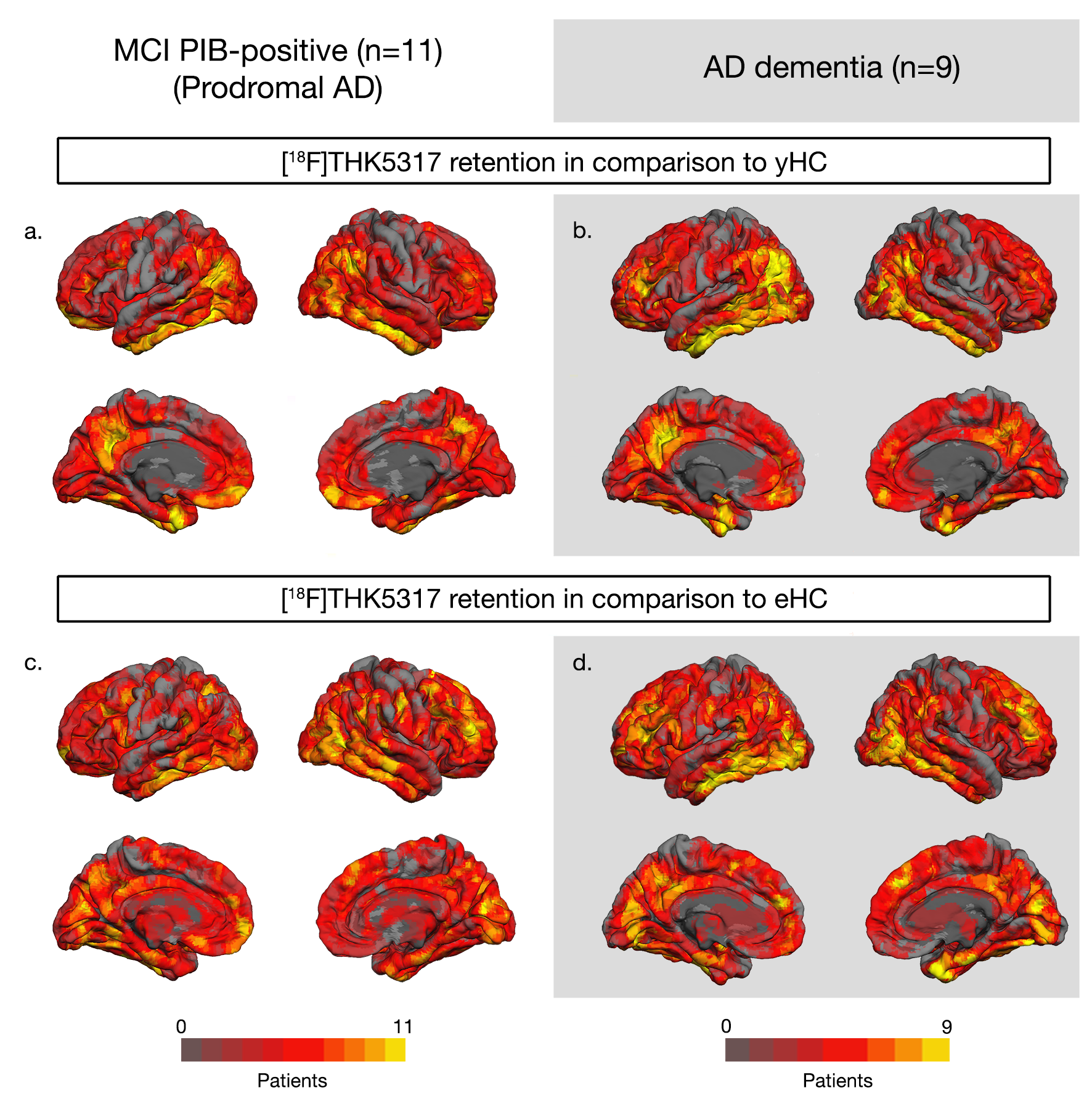


Online Resource 3. Regional patterns of abnormal [^18^F]THK5317 across all patients with prodromal Alzheimer’s disease (AD) (n=11) and AD dementia (n=9), in comparison to young healthy controls (yHC; A, B) and elderly healthy controls (eHC; C, D). Individual [^18^F]THK5317 DVR were compared to those from the control groups using z-score maps. Only voxels with z-score values above 1.96 (95% confidence interval) were considered. The resulting individual binarised images were summed to illustrate the areas of abnormal retention in the two diagnostic groups. Prodromal AD = mild cognitive impairment with [11C]PIB uptake above the normal range (threshold standard uptake value ratio of 1.41).

Online Resource 4. Regions with significant (p<0.001) negative voxel-wise correlations between [^18^F]THK5317 DVR retention and [^18^F]FDG SUVR uptake

| Regions of interest | [^18^F]THK5317 DVR vs [^18^F]FDG SUVR  negative correlations | | | |
| --- | --- | --- | --- | --- |
|  | Left hemisphere | | Right hemisphere | |
|  | Number of voxels | Max T value | Number of voxels | Max T value |
| Middle frontal gyrus | 25 | 4.2 | 93 | 5.2 |
| Lateral orbital gyrus | - | - | 31 | 4.3 |
| Lateral parietal lobe | 48 | 4.7 | - | - |
| Superior parietal gyrus | 24 | 4.3 | - | - |
| Posterior cingulate gyrus | 25 | 4.4 | - | - |

DVR = distribution volume ratio; SUVR = standard uptake value ratio.

Online Resource 5. Region-based correlations between [^18^F]THK5317 DVR and [^18^F]FDG SUVR, two by two across all Alzheimer’s disease patients (n=20). The analyses are illustrated in a correlation matrix-heatmap with only significant (p<0.05) correlations depicted. Occ: Occipital.

Online Resource 6. Regions with significant (p<0.001) positive voxel-wise correlations between [^18^F]THK5317 DVR and [^11^C]PIB SUVR retention

| Regions of interest | [^18^F]THK5317 DVR vs [^11^C]PIB SUVR  positive correlations | | | |
| --- | --- | --- | --- | --- |
|  | Left hemisphere | | Right hemisphere | |
|  | Number of voxels | Max T value | Number of voxels | Max T value |
| Hippocampus | 87 | 5.6 | 164 | 6.6 |
| Parahippocampal gyrus | - | - | 29 | 4.7 |
| Fusiform gyrus | 28 | 5.0 | - | - |
| Posterior Temporal lobe | 542 | 7.8 | 361 | 7.9 |
| Inferior frontal gyrus | 224 | 5.5 | - | - |
| Lateral orbital gyrus | 103 | 4.6 | - | - |
| Middle frontal gyrus | 136 | 4.9 | 313 | 4.8 |
| Superior frontal gyrus | 124 | 5.3 | 142 | 4.8 |
| Precentral gyrus | 178 | 5.1 | - | - |
| Postcentral gyrus | 87 | 5.1 | - | - |
| Lateral parietal lobe | 130 | 5.7 | 293 | 6.5 |
| Superior parietal gyrus | 576 | 7.1 | 1033 | 7.0 |
| Lateral occipital lobe | 429 | 7.9 | 216 | 5.8 |
| Lingual gyrus | 114 | 7.1 | 41 | 5.0 |
| Cuneus | 106 | 6.6 | 160 | 5.5 |
| Insula | 40 | 4.4 | - | - |
| Thalamus | 33 | 5.5 | 58 | 6.0 |

DVR = distribution volume ratio; SUVR = standard uptake value ratio.


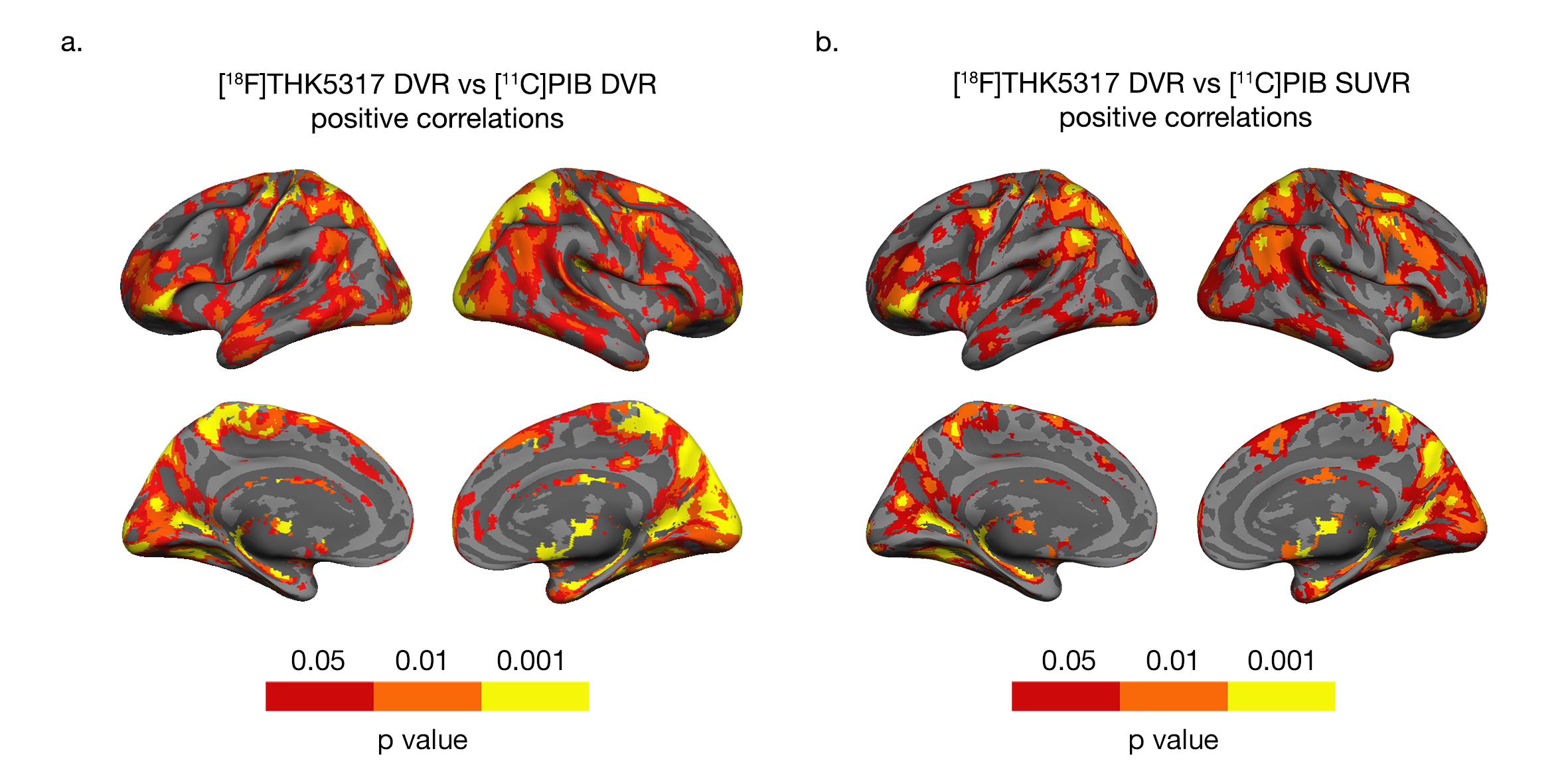


Online Resource 7. Voxel-based positive correlations between [^18^F]THK5317 DVR and [^11^C]PIB DVR retentions as well as [^18^F]THK5317 DVR and [^11^C]PIB SUVR retentions, two by two, across all Alzheimer’s disease patients (n=20). Three thresholds for statistical significance were applied (p<0.001, p<0.01 and p<0.05) as indicated. DVR = distribution volume ratio; SUVR = standard uptake value ratio.


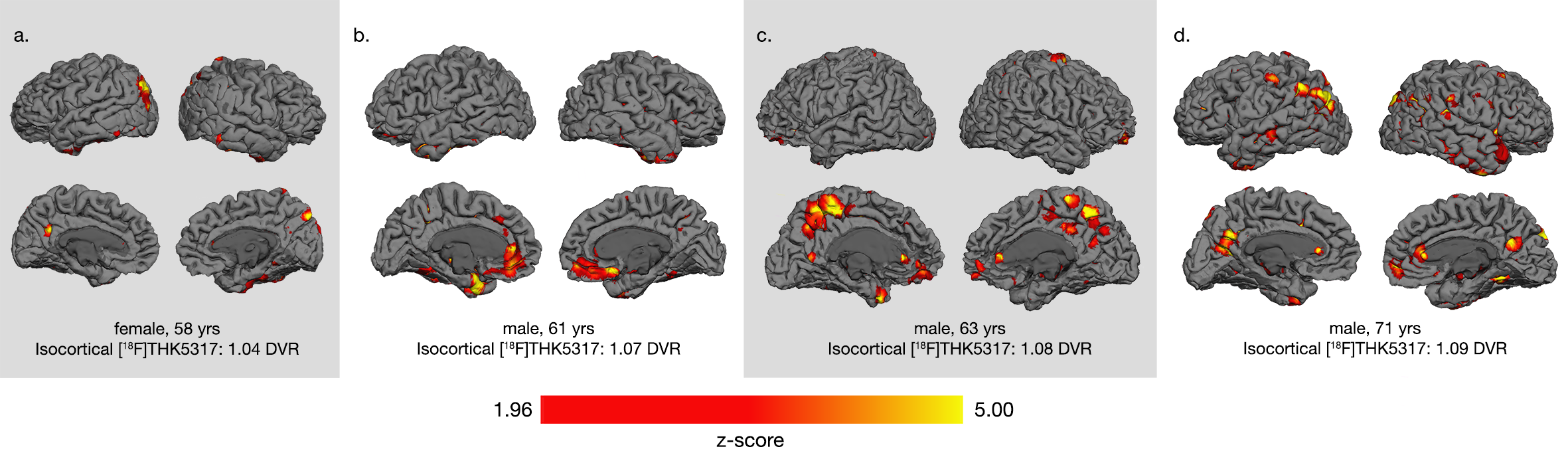


Online Resource 8. [^18^F]THK5317 DVR retention in the elderly healthy controls in comparison to young controls using z-score maps. A z-score threshold of 1.96 (95% confidence interval) was applied. The elderly healthy controls are ranked in order of increasing age.

Online Resource 9. Boxplots representing [^18^F]THK5317 DVR and SUVR retention before and after partial volume correction (PVC) across diagnostic groups in the hippocampus and the anterior cingulate gyrus. Filled dots represent outliers. Threshold for [^11^C]PIB positivity = 1.41. AD = Alzheimer’s disease; DVR = distribution volume ratio; eHC = elderly healthy controls; MCI = mild cognitive impairment; PIB = Pittsburgh compound B; Prodromal AD= MCI PIB-positive; SUVR = standard uptake value ratio; yHC = young healthy controls.
